# Temperature and water stress affect plant–pollinator interactions in *Borago officinalis* (Boraginaceae)

**DOI:** 10.1002/ece3.3914

**Published:** 2018-02-23

**Authors:** Charlotte Descamps, Muriel Quinet, Aurélie Baijot, Anne‐Laure Jacquemart

**Affiliations:** ^1^ Research Group « Genetics, Reproduction, Populations », Earth and Life Institute–Agronomy Université catholique de Louvain Louvain‐la‐Neuve Belgium

**Keywords:** *Borago officinalis*, flower rewards, high temperature, nectar, pollen, water stress

## Abstract

Climate change alters the abiotic constraints faced by plants, including increasing temperature and water stress. These changes may affect flower development and production of flower rewards, thus altering plant–pollinator interactions. Here, we investigated the consequences of increased temperature and water stress on plant growth, floral biology, flower‐reward production, and insect visitation of a widespread bee‐visited species, *Borago officinalis*. Plants were grown for 5 weeks under three temperature regimes (21, 24, and 27°C) and two watering regimes (well‐watered and water‐stressed). Plant growth was more affected by temperature rise than water stress, and the reproductive growth was affected by both stresses. Vegetative traits were stimulated at 24°C, but impaired at 27°C. Flower development was mainly affected by water stress, which decreased flower number (15 ± 2 flowers/plant in well‐watered plants vs. 8 ± 1 flowers/plant under water stress). Flowers had a reduced corolla surface under temperature rise and water stress (3.8 ± 0.5 cm^2^ in well‐watered plants at 21°C vs. 2.2 ± 0.1 cm^2^ in water‐stressed plants at 27°C). Both constraints reduced flower‐reward production. Nectar sugar content decreased from 3.9 ± 0.3 mg/flower in the well‐watered plants at 21°C to 1.3 ± 0.4 mg/flower in the water‐stressed plants at 27°C. Total pollen quantity was not affected, but pollen viability decreased from 79 ± 4% in the well‐watered plants at 21°C to 25 ± 9% in the water‐stressed plants at 27°C. Flowers in the well‐watered plants at 21°C received at least twice as many bumblebee visits compared with the other treatments. In conclusion, floral modifications induced by abiotic stresses related to climate change affect insect behavior and alter plant–pollinator interactions.

## 1 INTRODUCTION

Temperature and water stress are two abiotic constraints that natural systems face in the context of climate changes. Modifications in temperature and precipitation regimes affect plant and animal physiology, phenology, and, consequently, perturb the interactions between partners, such as between plants and their pollinators (Forrest, 2017; Gray & Brady, 2016; IPCC, 2014; Potts et al., 2010; Schweiger et al., 2010). To date, most studies investigating the effects of temperature and water stress on plant–pollinator interactions focus on plant and insect phenology and distribution (Bartomeus et al., 2011; González‐Varo et al., 2013; Hegland, Nielsen, Lázaro, Bjerknes, & Totland, 2009; Settele, Bishop, & Potts, 2016). But few studies consider the effects of these abiotic constraints on plant–pollinator interactions via plant physiological processes, although modifications at the plant level could have consequences for floral traits and flower‐reward availability (Mu et al., 2015; Parmesan & Hanley, 2015; Scaven & Rafferty, 2013; Takkis, Tscheulin, Tsalkatis, & Petanidou, 2015).

High temperatures and water stress alter many physiological processes during the plant life cycle and affect plants at the molecular, cellular, and organismal level (Barnabás, Jäger, & Fehér, 2008; Hedhly, 2011; Pandey, Ramegowda, & Senthil‐Kumar, 2015; Wahid, Gelani, Ashraf, & Foolad, 2007). Increases in temperature induce heat stress when temperatures suddenly increase above the optimal growth temperature, causing stressful conditions and having negative consequences on vegetative growth (Prasad, Staggenborg, & Ristic, 2008). The effect of these abiotic stresses depends on the duration and intensity of the stress (Wahid et al., 2007; Zinn, Tunc‐Ozdemir, & Harper, 2010). High temperature and water stress can produce common or specific effects (Pandey et al., 2015), the combination of both stresses can have a stronger effect on plants than each stress alone (Barnabás et al., 2008; Rizhsky, Liang, & Mittler, 2002). Both stresses lead to a reduction in photosynthesis (Pandey et al., 2015). Water stress leads to stomatal closure, reducing water potential and CO_2_ uptake, thus leading to inhibition of photosynthesis (Barnabás et al., 2008; Khan, Paull, Siddique, & Stoddard, 2010; Prasch & Sonnewald, 2015). High‐temperature stress damages Photosystem II (PSII) (Prasad et al., 2008). The combination of the two stresses has deleterious effects on photosynthetic activity (Pandey et al., 2015; Prasch & Sonnewald, 2015; Rizhsky et al., 2002). Regarding vegetative growth, high temperature and water stress have mainly contrasting effects (Pandey et al., 2015). Plants tolerate water stress by reducing water loss and increasing water uptake, by reducing leaf number and leaf area, and by increasing root growth (Berger, Palta, & Vadez, 2016; Connor & Jones, 1985). Plants tolerate high temperatures by increasing their transpiration rate, including by increasing leaf number and area (Prasad et al., 2008). Under temperature rise, plant height can be reduced (Qaderi, Kurepin, & Reid, 2012). Both stresses affect therefore vegetative traits.

In addition to an indirect effect via inhibition of photosynthesis, high temperature and water stress also directly affect reproductive growth which is even more sensitive to abiotic constraints than the vegetative growth (Hedhly, 2011; Prasad et al., 2008; Snider & Oosterhuis, 2011; Zinn et al., 2010). These abiotic stresses can cause floral bud abortion and reduce flower number, leading to decreased reproductive success (Morrison & Stewart, 2002; Muhl, du Toit, Steyn, & Apostolides, 2013). They can also affect the size of floral organs (Carroll, Pallardy, & Galen, 2001; Koti, Reddy, Reddy, Kakani, & Zhao, 2005; Sato et al., 2006). These abiotic stresses, if they occur during flowering, limit flower rewards. High temperature and water stress affect nectar production through changes in nectar volume and sugar concentration. Nectar volume generally increases with increasing temperature, up to a species‐specific optimum temperature (Nicolson & Susan, 2007). Above this optimum temperature, nectar volume decreases (Mu et al., 2015). In response to water stress, nectar volume generally decreases (Carroll et al., 2001). Temperature rise and water stress seem to have less effect on sugar concentration (Carroll et al., 2001; Mu et al., 2015). They affect also pollen development and viability, which could perturb fertilization and seed development (Barnabás et al., 2008; Hedhly, 2011; Snider & Oosterhuis, 2011). Such modifications in pollen production mainly involve the following: (1) a reduction in the numbers of mature pollen grains; (2) abnormal pollen development, leading to decreased viability and germination capacity; and (3) abnormal anther morphology, leading to reduced pollen transfer (Bishop, Potts, & Jones, 2016; Devasirvatham et al., 2012; Hedhly, 2011; Sage et al., 2015). Both stresses applied during reproductive development lead to a reduction in fruit and seed sets (Hedhly, 2011; Pandey et al., 2015).

The modifications of floral traits and flower rewards due to temperature rise and water stress could have consequences for plant–pollinator interactions because flowers and flower rewards are key elements affecting pollinator abundance and activity (Potts, Vulliamy, Dafni, Ne'Eman, & Willmer, 2003). Nectar provides the main sugar source for insect pollinators (Nicolson & Susan, 2007). The volume and total sugar concentration determine the energetic value of the nectar for insects (Cnaani, Thomson, & Papaj, 2006; Nicolson & Thornburg, 2007). Pollen is the principal source of polypeptides, amino acids, and phytosterols (Cane, 2016; Hanley, Franco, Pichon, Darvill, & Goulson, 2008; Roulston, Cane, & Buchmann, 2000). Flower rewards with higher quality (high sugar content in nectar, high polypeptide content in pollen) and quantity are more attractive to pollinators (Bailes, Ollerton, Pattrick, & Glover, 2015; Cartar, 2004; Kudo & Harder, 2005; Somme et al., 2014; Zhao, Lu, & Conner, 2016), whereas rewards with lower quality or quantity are less attractive (i.e., reduced pollinator abundance and activity) (Larsson & Franzén, 2007; Wallisdevries, Van Swaay, & Plate, 2012). Therefore, modifications of floral traits and flower rewards might alter the attractiveness of flowers to pollinators (Forrest, 2017; Scaven & Rafferty, 2013). Such modifications to plant–pollinator interactions affect both partners (Roger, Michez, Wattiez, Sheridan, & Vanderplanck, 2016; Roger et al., 2017; Scaven & Rafferty, 2013).

The aim of this study was to understand how floral traits and flower rewards could be affected by temperature rise and water stress through plant physiological processes and, eventually, how insects modify visitation behavior. Our hypothesis was that increases in temperature and drought affect plant growth and physiology, leading to decreased flower‐reward production; reduction in flower rewards will modify insect visitation behavior. We tested this hypothesis on an entomophilous species, *Borago officinalis*, that is broadly distributed and attractive to insects. We addressed three questions: (1) Do temperature and water stress interact in their effects on plant functions? (2) Do these stresses influence directly floral traits and flower rewards or is this response mediated through influence on growth and physiology? (3) Do these stresses modify flower visitation by insects?

## 2 MATERIAL AND METHODS

### 2.1 Plant material and growth conditions

*Borago officinalis* is an annual, entomophilous plant (60–90 cm), with a large floral display. Its native range is the Mediterranean region. The flowering period extends from June to September, and about hundred flowers are produced per plant. Flowers are grouped in terminal inflorescences that form scorpioid cymes. Flowers are hermaphroditic, 5‐merous, and actinomorphic. Petal color changes from pink to blue during anthesis that lasts about 3 days. *B. officinalis* is mainly pollinated by bumblebees and honeybees, which represent 80% of insect visits (Thom et al., 2016; A. Baijot, pers. obs.).

*Borago officinalis* seeds were provided by Vilmorin nursery (Saint‐Quentin, France). Seeds were placed in a germination chamber (Economic Delux model ECD01E; Snijders Scientific, Tilburg, Netherlands) under 20°C/18°C day/night temperature and a 16‐hr light (L):8‐hr dark (D) photoperiod. Seedlings at the three‐leaf stage were transplanted into 2‐L pots filled with a 1:1 (v/v) mix of sand (size 0/5, M PRO, Netherlands) and universal peat compost (DCM, Amsterdam, Netherlands), and grown in the glasshouse at the University campus (Louvain‐la‐Neuve 50°39′58″N; 4°37′9″E, Belgium). They were watered daily with rainwater until the beginning of the experiment. Treatments were applied at floral transition, 4 weeks after sowing. At this stage, bolting occurred, flowering stem developed, and the first floral buds were visible. Plants were subjected to three temperature regimes (21, 24, and 27°C) and two watering regimes (watering vs. water stress) to investigate the main effects of temperature and water stresses and their interactions. In total, six treatments were applied to 13 plants per treatment: 21°C well‐watered (21WW), 21°C water‐stressed (21WS), 24°C well‐watered (24WW), 24°C water‐stressed (24WS), 27°C well‐watered (27WW), and 27°C water‐stressed (27WS). In total, 78 plants were monitored in three growth chambers under three temperature regimes (day/night): 21/19°C, 24/22°C, and 27/25°C. Photoperiod was 16L:8D, and relative humidity was maintained at 80 ± 10%. Light was supplied by Philips HPIT 400 W lamps (Philips Lighting S.A., Brussels, Belgium), and light irradiance was at 155 ± 20 μmol m^−2^ s^−1^ at canopy level (Skye Instruments Quantum Sensor quantum meter; Hansatech Instruments, Norfolk, UK). Each growth chamber was divided into two parts to accommodate two watering regimes. The well‐watered plants received daily watering (soil humidity about 30%), whereas the water‐stressed plants were watered twice a week (soil humidity lower than 15%). Growth chamber experiments lasted 6 weeks. Water stress was applied after 1 week of acclimation to the growth chambers (this week was considered week 0). Soil water content was quantified with a ProCheck sensor handheld reader (Decagon Devices, Pullman, USA). Experiments were repeated twice on consecutive years.

### 2.2 Vegetative trait measurements

The number of nodes and leaves were counted once a week on 10 plants per treatment during the 6‐week experiment. The number of nodes on the main stem was counted as soon as the flowering stem developed. Green leaves (>2 cm), floral buds (>0.5 cm), and flowers at anthesis were quantified per node separately on the main stem and on branched shoots, called ramifications. Stem and ramification lengths were measured at the end of the experiment (i.e., 5 weeks after the stress induction), between the first and last nodes.

### 2.3 Physiological parameters

Physiological measurements were performed on the 5th‐node leaf of 10 plants per treatment, between 10 a.m. and 3 p.m., at the beginning of the experiment, and 2 and 4 weeks after inducing stress. The measured parameters were chlorophyll fluorescence, chlorophyll content, stomatal conductance (g_s_), and gas exchange.

Chlorophyll fluorescence was monitored using a fluorescence monitoring system fluorometer (FMS II; Hansatech Instruments, Norfolk, UK). The quantified parameter was PSII efficiency (ΦPSII), which measures the proportion of light absorbed by PSII that is used in photochemistry (Maxwell & Johnson, 2000). Leaf portions were dark‐adapted for 30 min before illumination with a first pulse at 18,000 mmol m^−2^ s^−1^ followed by a constant level of actinic light (660 mmol m^−2^ s^−1^) for 2 min. A second saturating pulse of 18,000 mmol m^−2^ s^−1^ was subsequently applied. Chlorophyll content index (CCI) was measured using a chlorophyllometer (Opti‐Sciences, CCM‐200), and the measurement was taken three times on the same leaf. An automatic porometer (AP4 System, Delta‐T Devices) was used to measure g_s_ on the abaxial surface of the leaf.

Gas exchanges (instantaneous photosynthetic (A_i_,) and transpiration rate (E_i_)) were measured using an infrared gas analyzer (IRGA ADC BioScientific LCI‐SD system, serial No.33413, Hoddesdon, UK). Temperature and relative humidity in the cuvette were set at 21, 24, or 27°C according to the growth chamber and 70 ± 5%, respectively. Instantaneous water use efficiency (WUE) was calculated as WUE = A/E.

### 2.4 Floral trait and flower reward measurements

The numbers of floral buds (>0.5 cm) and flowers at anthesis were counted once a week on 10 plants per treatment during the 6‐week experiment. They were quantified per node separately on the main stem and on ramifications. The petal length was measured on 10 random flowers from separate plants per treatment once a week. Petal length was measured as the length between nectaries and petal apex. Moreover, 3 weeks after inducing stress, flowers were dissected, and all organs were separated and scanned. Their dimensions were estimated by scan analysis using ImageJ software. During the experiment, modifications of flower shape and/or morphology were observed, and abnormal flowers were counted.

One anther per flower was collected from six random flower buds per treatment (from different individuals) one day before anthesis and stored in FAA solution (70% ethanol, glacial acid acetic, 35% formaldehyde; 18:1:1). To count the number of pollen grains per anther, anthers were crushed separately and placed in microfuge tubes containing 50 μl Alexander's stain. Tubes were then vortexed to disperse pollen grains in the solution. A subsample of 1 μl was used to count all pollen grains on a microscope slide under a light microscope (Nikon Eclipse E400, G 400x). Counts were performed in triplicate for each anther and were performed 3 weeks after induction of the stress. Pollen viability was assessed using fluorescein diacetate (Dafni, Kevan, & Husband, 2005). Five flowers per treatment were randomly collected a few hours after their opening; one anther per flower was removed and added to fluorescein diacetate solution. Pollen viability was determined using a minimum of 200 pollen grains per sample. Counts were performed in triplicate for each anther. Pollen viability was estimated 3 weeks after induction of the stress.

Nectar was extracted with 10‐μl glass capillary tubes (Hirschmann Laborgeräte, Eberstadt, Germany) from five flowers per treatment (flowers from five different plants). Total sugar concentration (C, g sucrose/100 g solution) was measured with a low‐volume hand refractometer (Eclipse handheld refractometer; Bellingham and Stanley, Tunbridge Wells, UK). Nectar sugar content per flower (*s*, mg) was calculated as *s* = 10 × *d* × *v* × *C*, where d is the density of a sucrose solution at concentration *C* (*d* = 0.0037921 × *C* + 0.0000178 × *C* ^2^ + 0.9988603) and *v* is nectar volume (ml) (Prys‐Jones & Corbet, 1991).

### 2.5 Insect visitor observations

Five weeks after induction of stress, three plants per treatment (with 8–12 blue flowers per plant) were exposed outside to insect visitors at the University experimental garden, on 4 m^2^ observation plots. Plants were identified according to their previous stress treatment. Bumblebee visits were recorded during sunny days (between 10 a.m. and 4 p.m., 25°C on average). In total, we followed 33 bumblebee individuals for a total of 1,148 flower visits over 164 min. Individuals were followed during all their visits to flowers in the observation plots. During tracking, relative position of the successive visited plants, plant identity, and the number of flowers visited per plant were recorded. For each plant treatment, visitation rate was extrapolated as the number of visited flowers per plant during 60 min divided by the number of flowers on the plant. The mean number of visited flowers per plant and per bumblebee was also compared among stress treatments.

### 2.6 Statistical analyses

Normality of the data was estimated using QQ plots and Shapiro–Wilk test. Linear mixed models and analysis of variance (type II) were performed to a significance level of *p *<* *.05 to evaluate the effects of temperature rise and water stress. For repeated measurements on the same plant at a time point (chlorophyll content measurements, pollen number, and viability), linear mixed models were made with two fixed factors and their interaction (temperature × water) and plants as the repeated factor. Linear mixed models were made with three fixed factors (temperature × water × week) and plants as the repeated factor, to analyze repeated measurements over time on the same plants (number of stem leaves, ramification leaves, and open flowers). Analysis of variance was performed to analyze data at each time point. Tukey's test was performed for post hoc analyses. Chi‐squared test was used to compare proportions of ramifications under and above the first node to the flower. To identify correlations between physiological, vegetative, and floral biology parameters, principal component analysis (PCA) and Pearson correlation plots were performed. All analyses were performed with R 3.2.1, using package *car* for *F* test, package *lme4* for linear mixed models, package *FactomineR* for PCA, and package *corrplot* for correlations. Data are presented as means ± standard errors (*SE*).

## 3 RESULTS

### 3.1 Vegetative traits

**Figure 1:**
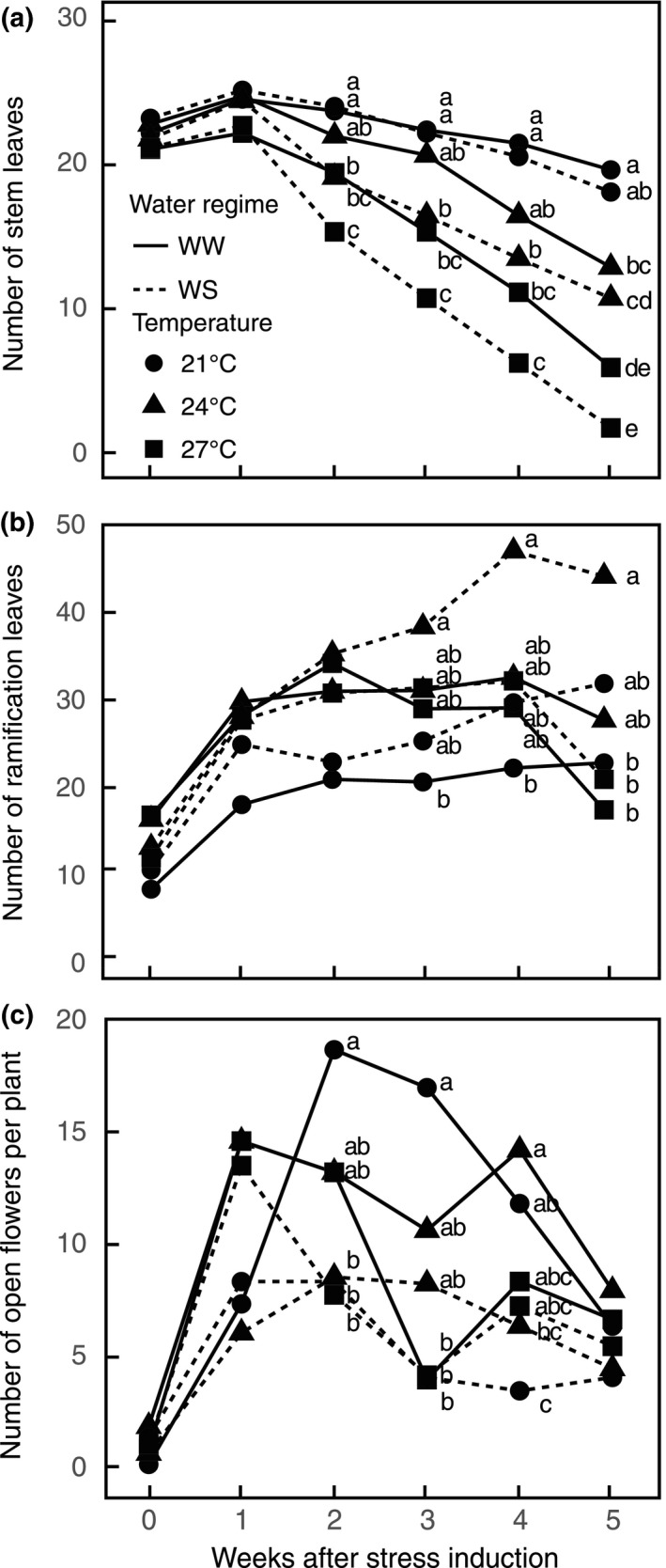
Effects of temperature rise and water stress on the evolution of (a) number of leaves on the main stem; (b) number of leaves on the ramifications; (c) number of open flowers per plant. *N *=* *10. Data are means. Data points followed by different letters are significantly different at *p *<* *.05 among treatments at a time point. WW, well‐watered; WS, water‐stressed

The length and the number of nodes on the main stem depended on the temperature (Table 1). The length of main stem was the highest at 24°C and lowest at 27°C. The number of nodes was also the lowest at 27°C. The final number of leaves on the main stem decreased with both temperature rise (*F*_2,54_ = 43.98, *p* < .001) and water stress treatments (*F*_1,54_ = 4.02, *p* = .05, Figure 1a).

**Table 1:** Effects of temperature rise and water stress on vegetative traits, 5 weeks after induction of stress

Treatment_1_	Length of main stem (cm)	Nodes on main stem	Number of ramifications
21WW	56.8 ± 2.9^ab^	22.8 ± 0.9^ab^	5.2 ± 0.5^c^
21WS	52.1 ± 1.9^b^	24.2 ± 1^a^	8.2 ± 0.8^abc^
24WW	64.7 ± 4^a^	22.1 ± 0.8^ab^	7.7 ± 0.7^abc^
24WS	57.9 ± 2.1^ab^	23.4 ± 0.9^ab^	10.7 ± 0.7^a^
27WW	49.3 ± 1.8^b^	20.8 ± 0.6^b^	7.4 ± 0.6^bc^
27WS	51.3 ± 2.3^b^	20.8 ± 0.6^b^	9.1 ± 0.9^ab^
Temp._2_	*F*_2,54_ = 9.13; *p* < .001	*F*_2,54_ = 5.97; *p* = .005	*F*_2,54_ = 6.17; *p* = .003
Water	*F*_1,54_ = 2.22; *p* = 0.14	*F*_1,54_ = 1.87; *p* = .18	*F*_1,54_ = 19.15; *p* < .001
Temp. × Water	*F*_2,54_ = 1.56; *p* = .22	*F*_2,54_ = 0.47; *p* = .63	*F*_2,54_ = 0.55; *p* = .58

^1^ *N* = 10. Data are means ± *SE*. Data points followed by different letters for each parameter are significantly different at *p* < .05 among treatments. 21 = 21°C; 24 = 24°C; 27 = 27°C; WW, well‐watered; WS, water‐stressed.

^2^Two‐way ANOVA results, testing for the main and interactive effects of temperature (Temp.) and water treatments.

The development of ramifications was stimulated by both temperature rise and water stress (Table 1). Plants branched the most in the 24WS treatment. Similarly, the number of leaves on the ramifications (*F*_2,54_ = 8.41, *p *<* *.001) was significantly higher for this treatment, compared to those at 21°C (Figure 1b). Most of the ramifications (71 ± 7%) developed below the first inflorescence (χ^2^ = 12.43, *df* = 5, *p *=* *.029), regardless of treatment. As a result at 27°C, most of the remaining leaves were ramification leaves (Temp: *F*_2,54_ = 12.24, *p *<* *.001; Water: *F*_1,54_ = 2.88, *p *=* *.09). Ramification development discriminated the water‐stressed treatments, particularly the 24WS treatment.

### 3.2 Photosynthesis‐related parameters

Physiological parameters were more affected by temperature than by water stress (Table 2). Regarding photosynthesis, chlorophyll content of leaves decreased by more than 40% at 27°C compared to 21°C and 24°C. ΦPSII and the maximum potential quantum efficiency of PSII (Fv/Fm; *F*_2,52_ = 9.35, *p* < .001, data not shown) were reduced at 27°C. However, A_i_ increased with increasing temperature. Concerning gas exchange, g_s_ was significantly higher at 24°C compared to the other temperatures. Both temperature rise and water stress affected E_i_; E_i_ was higher at 24°C and decreased with water stress. Because of A_i_ and E_i_, the lowest instantaneous WUE was observed at 24°C and the highest at 27°C. The instantaneous WUE increased with water stress.

**Table 2:** Effects of temperature rise and water stress on chlorophyll content, chlorophyll fluorescence parameters, and gas exchange 2 weeks after stress induction

Treatment_1_	Chlorophyll content (−)	ΦPSII (−)	g_s_ (mmol m^−2^ s^−1^)	A_i_ (μmol m^−2^ s^−1^)	E_i_ (mmol m^−2^ s^−1^)	WUE (A/E) (μmol CO_2_ mmolH_2_O^−1^)
21WW	21.9 ± 1.9^a^	0.82 ± 0.01^a^	58 ± 4^c^	1 ± 0.19^ab^	1.07 ± 0.05^bc^	0.95 ± 0.25^ab^
21WS	19 ± 3.2^ab^	0.82 ± 0.01^a^	32 ± 3^c^	0.74 ± 0.13^b^	0.53 ± 0.06^c^	1.36 ± 0.24^a^
24WW	23.4 ± 3^a^	0.79 ± 0.01^ab^	265 ± 64^a^	0.96 ± 0.14^b^	2.03 ± 0.21^a^	0.47 ± 0.1^b^
24WS	20.9 ± 2.2^ab^	0.79 ± 0.01^ab^	212 ± 33^ab^	1.37 ± 0.38^ab^	1.42 ± 0.19^ab^	0.86 ± 0.23^ab^
27WW	13.2 ± 2^ab^	0.75 ± 0.02^b^	109 ± 14^bc^	2.01 ± 0.32^a^	1.62 ± 0.2^ab^	1.27 ± 0.23^a^
27WS	11.3 ± 2.6^b^	0.75 ± 0.02^b^	73 ± 12^c^	1.36 ± 0.23^ab^	1.01 ± 0.15^bc^	1.39 ± 0.26^a^
Temp._2_	*F*_2,54_ = 8.95; *p* < .001	*F*_2,52_ = 11.48; *p* < .001	*F*_2,54_ = 22.35; *p* < .001	*F*_2,54_ = 5.56; *p* = .006	*F*_2,54_ = 17.68; *p* < .001	*F*_2,54_ = 9.10; *p* < .001
Water	*F*_1,54_ = 1.46; *p* = .23	*F*_1,52_ = 0.01; *p* = .92	*F*_1,54_ = 2.39; *p* = .13	*F*_1,54_ = 0.58; *p* = .45	*F*_1,54_ = 20.72; *p* < .001	*F*_1,54_ = 5.48; *p* = .023
Temp. × Water	*F*_2,54_ = 0.02; *p* = .97	*F*_2,52_ = 0.01; *p* = .99	*F*_2,54_ = 0.11; *p* = .9	*F*_2,54_ = 2.26; *p* = .11	*F*_2,54_ = 0.02; *p* = .98	*F*_2,54_ = 0.49; *p* = .62

ΦPSII, Photosystem II efficiency; g_s_, stomatal conductance; A_i_, instantaneous photosynthetic rate; E_i_, instantaneous transpiration rate; WUE, water use efficiency.

^1^ *N* = 10. Data are means ± *SE*. Data points followed by different letters for each parameter are significantly different at *p* < .05 among treatments. 21 = 21°C; 24 = 24°C; 27 = 27°C; WW, well‐watered; WS, water‐stressed,

^2^Two‐way ANOVA results, testing for the main and interactive effects of temperature (Temp.) and water treatments.

### 3.3 Floral traits

The number of floral buds decreased with increasing temperature (Table 3). Furthermore, more than 50% of the floral buds aborted on water‐stressed plants so that the number of open flowers was lower for these treatments compared to well‐watered plants (Figure 1c). Differences in the number of open flowers between water‐stressed and well‐watered plants were particularly visible 2 weeks after induction of stress. On average, eight flowers were open at the same time per water‐stressed plant compared to 15 flowers per well‐watered plant. Three weeks after induction of stress, flower production decreased in all treatments, especially 27WW. The pattern of flower production along the main stem was not significantly modified by temperature rise (*F*_2,54_ = 1.62, *p *=* *.21) or water stress (*F*_1,54_ = 3.16, *p *=* *.08).

**Table 3:** Effects of temperature rise and water stress on parameters related to floral biology 3 weeks after stress induction

Treatment_1_	Number of floral buds	Petal length (cm)	Corolla surface area (cm^2^)	Abnormal flowers (%)
21WW	89 ± 4^a^	1.69 ± 0.05^a^	3.77 ± 0.45^a^	1.3 ± 0.5^b^
21WS	70 ± 9^ab^	1.51 ± 0.04^ab^	3.67 ± 0.11^a^	4.1 ± 2^ab^
24WW	60 ± 6^ab^	1.64 ± 0.03^a^	3.22 ± 0.08^ab^	6.6 ± 2.3^ab^
24WS	69 ± 10^ab^	1.38 ± 0.06^b^	2.69 ± 0.18^ab^	4.3 ± 2.2^ab^
27WW	57 ± 6^b^	1.60 ± 0.04^a^	3.09 ± 0.42^ab^	10.7 ± 2.2^a^
27WS	50 ± 7^b^	1.38 ± 0.06^b^	2.23 ± 0.07^b^	10.1 ± 2.7^a^
Temp._2_	*F*_2,54_ = 6.44; *p *=* *.003	*F*_2,54_ = 2.7; *p *=* *.08	*F*_2,24_ = 8.11; *p *=* *.002	*F*_2,54_ = 6.86; *p *=* *.002
Water	*F*_1,54_ = 0.9; *p *=* *.34	*F*_1,54_ = 28.69; *p *<* *.001	*F*_1,24_ = 5; *p *=* *.03	*F*_1,54_ = 0.002; *p *=* *.96
Temp. × Water	*F*_2,54_ = 1.92; *p *=* *.15	*F*_2,54_ = 0.39; *p *=* *.68	*F*_2,24_ = 0.99; *p *=* *.39	*F*_2,54_ = 0.76; *p *=* *.47

^1^ *N* = 10. Data are means ± *SE*. Data points followed by different letters for each parameter are significantly different at *p* < .05 among treatments. 21 = 21°C; 24 = 24°C; 27 = 27°C; WW, well‐watered; WS, water‐stressed.

^2^Two‐way ANOVA results, testing for the main and interactive effects of temperature (Temp.) and water treatments.

Temperature rise and water stress affected flower morphogenesis and shape. Petal length decreased with water stress (Table 3) but not with temperature rise, while corolla surface area (Table 3) decreased with both temperature rise and water stress. It was reduced by 40% in 27WS compared to 21WW. Three weeks after induction of stress, flower abnormalities were observed in response to temperature rise, particularly at 27°C (Table 3).

### 3.4 Flower rewards

**Figure 2:**
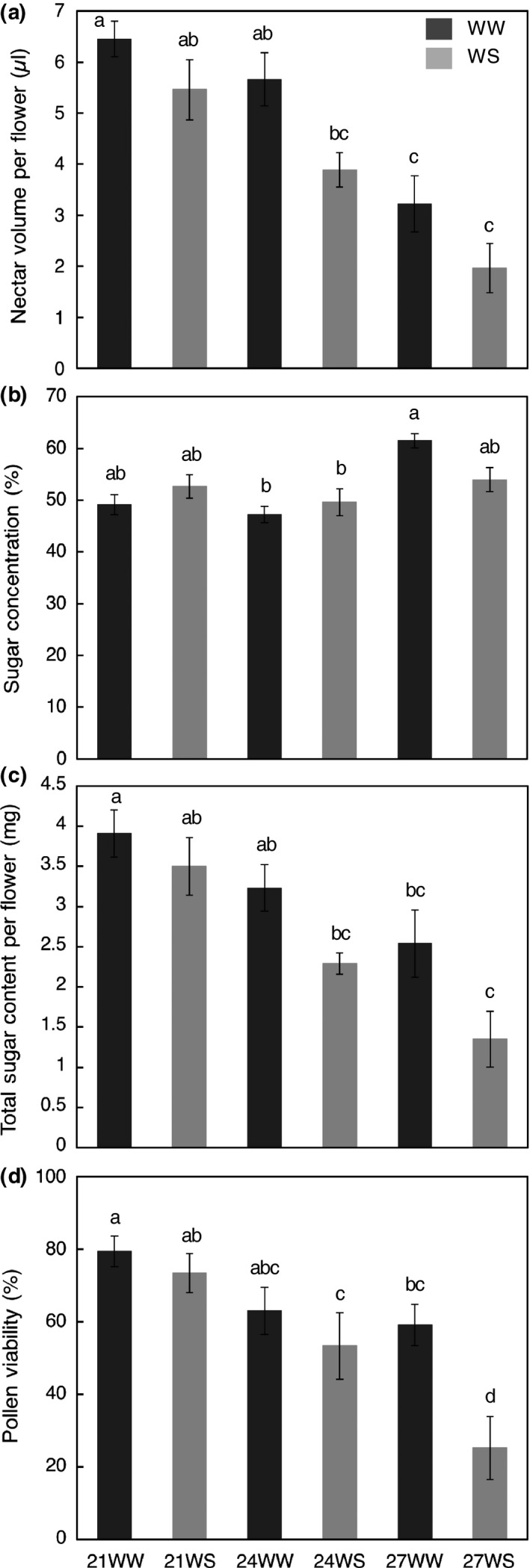
Impacts of temperature rise and water stress on flower‐reward production 3 weeks after stress induction: (a) nectar volume per flower (μl); (b) sugar concentration in nectar (%); (c) total sugar content in nectar per flower (mg); (d) pollen viability (%). *N *=* *10 plants. Data are means ± *SE*. Treatments followed by different letters are significantly different at *p *<* *.05. 21 = 21°C; 24 = 24°C; 27 = 27°C; WW, well‐watered; WS, water‐stressed

Nectar volume decreased with both temperature rise (F_2,52_ = 25.06, *p *<* *.001) and water stress (*F*_2,52_ = 11.69, *p *=* *.001, Figure 2a). As a result, nectar volume decreased by 70% under 27WS compared to 21WW. Sugar concentration was affected by temperature rise (*F*_2,52_ = 11.25, *p *<* *.001) but not water stress (*F*_1,52_ = 0.18, *p *=* *.68, Figure 2b); their effects varied according to treatment (*F*_2,52_ = 4.31, *p *<* *.001). The highest sugar concentration was recorded at 27°C. This high sugar concentration did not balance the low nectar volume observed for this temperature; total sugar content per flower was four times lower at 27°C than at 21°C. As with nectar volume, total sugar content of nectar decreased with both temperature rise (*F*_2,52_ = 14.96, *p *<* *.001) and water stress (*F*_2,52_ = 10.67, *p *=* *.002, Figure 2c).

The total number of pollen grains per anther was not significantly affected by temperature rise (*F*_2,29_ = 1.86, *p *=* *.17) or water stress (*F*_1,29_ = 0.0004, *p *=* *.98) and was on average 12,700 ± 4,800. However, pollen viability was significantly reduced by temperature rise (*F*_2,29_ = 12.96, *p *<* *.001) and water stress (*F*_1,30_ = 7.83, *p *=* *.009, Figure 2d). The pollen viability was about 80% at 21°C and dropped to <60% at 27°C. The impact of water stress was mainly observed at 27°C where pollen viability decreased by 50% between well‐watered and water‐stressed plants.

### 3.5 Flower visitation by bumblebees

**Figure 3:**
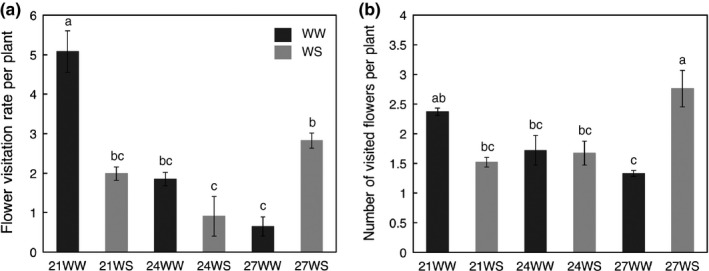
Impacts of temperature rise and water stress on bumblebee visits (*N* visits = 1,148): (a) flower visitation rate per plant expressed as the number of visits per flower per hour; (b) number of visited flowers per plant expressed as the number of flowers visited per bumblebee individual before it moved to the next plant. Data are means ± *SE*. Treatments followed by different letters are significantly different at *p *<* *.05. 21 = 21°C; 24 = 24°C; 27 = 27°C; WW, well‐watered; WS, water‐stressed

Regardless of treatment, an individual bumblebee visited on average 35 flowers in 5 min. Flower visitation rate was affected by temperature rise (*F*_2,12_ = 17.56, *p *<* *.001) depending on watering regime (interaction effect of Temp. × Water, *F*_2,12_ = 23.01, *p *<* *.001) and was higher for 21°C plants than for the other plants (Figure 3a). For plants grown at 21°C, 21WW plants were more visited than 21WS plants, while for plants grown at 27°C, there were more visits to 27WS plants than to 27WW. The number of visited flowers per plant before moving to the next plant also depended on the combination between temperature and water regime (interaction effect of Temp. × Water, *F*_2,12_ = 19.34, *p *=* *.002; Figure 3b); water stress decreased the number of visited flowers at 21°C and increased it at 27°C. In general, bumblebees visited more flowers per plant before moving to the next plant on 27WS plants compared to the other treatments.

### 3.6 Principal component and correlation analyses

**Figure 4:**
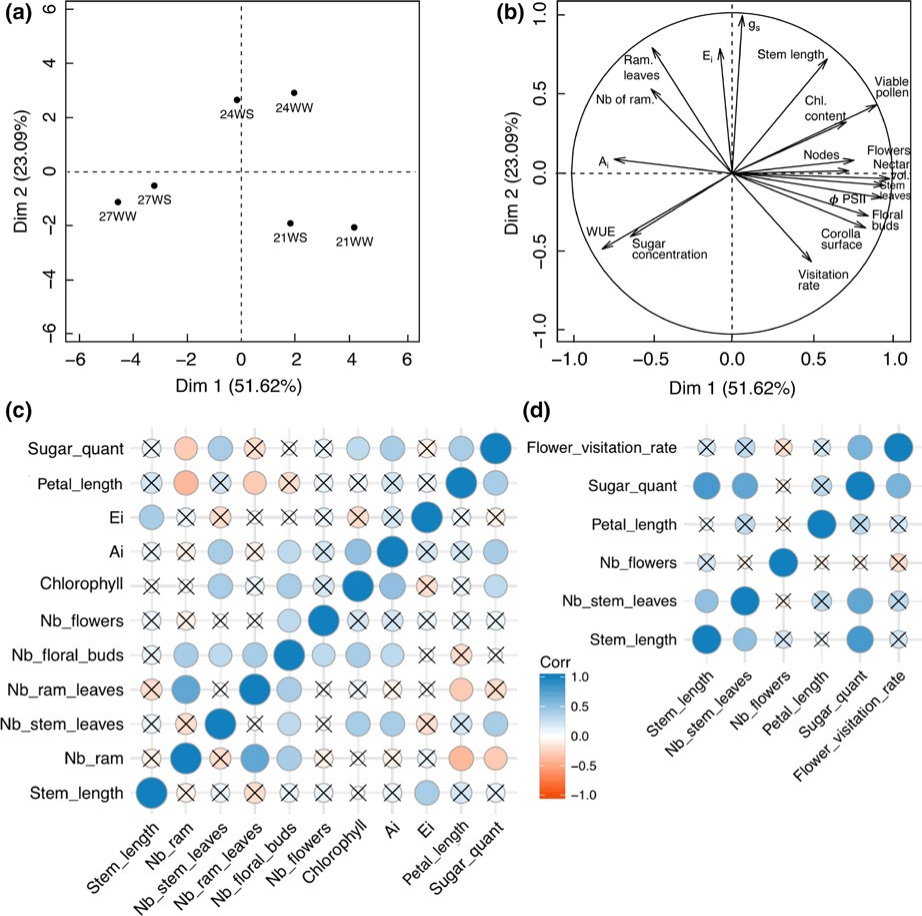
Principal component analysis (PCA) (a, b) and correlation graphs (c, d) of vegetative, physiological, floral parameters, and flower visitation rate in response to temperature rise (21, 24, and 27°C) and water stress treatments (WW, well‐watered plants; WS, water‐stressed plants). (a) Individual graph; (b) variable graph of PCA 3 weeks after stress induction; (c) correlations between physiological, vegetative, and floral parameters of all plants (*N* = 60); and (d) correlations between vegetative and floral parameters, and insect visitation of plants exposed to bumblebees (*N* = 18) 5 weeks after stress induction (A_i_, instantaneous photosynthetic rate; Chl, chlorophyll; E_i_, instantaneous transpiration rate; g_s_, stomatal conductance; nb, number; ram, ramification; sugar_quant, nectar sugar quantity per flower; WUE, water use efficiency; ΦPSII, Photosystem II efficiency). Nonsignificant correlations (*p *<* *.05) are marked with a cross

The PCA showed that 75% of the variance was explained by principal component 1 (Axis 1) and principal component 2 (Axis 2) (Figure 4a,b). The parameters were more discriminated by temperature rise than by water stress. Axis 1 highlighted the differences between 21°C and 27°C and separated the two treatments based on the number of leaves on the main stem, the efficiency of PSII, the number of floral buds and flowers, the corolla surface, the nectar volume, and the pollen viability that were the highest at 21°C and WUE that was the highest at 27°C. Axis 2 discriminated 24°C from the other temperatures due to higher values of the physiological parameters (g_s_ and E_i_), stem length, and ramification development.

As shown in Figure 4c, the nectar sugar quantity was positively correlated with the development of the main stem, the photosynthetic parameters (chlorophyll content, A_i_), and the flower size, while it was negatively correlated with the development of ramifications. The flower visitation rate was mainly correlated with the amount of sugars in the nectar (Figure 4d).

## 4 DISCUSSION

### 4.1 Do temperature and water stress interact with their effect on plant functions?

**Figure 5:**
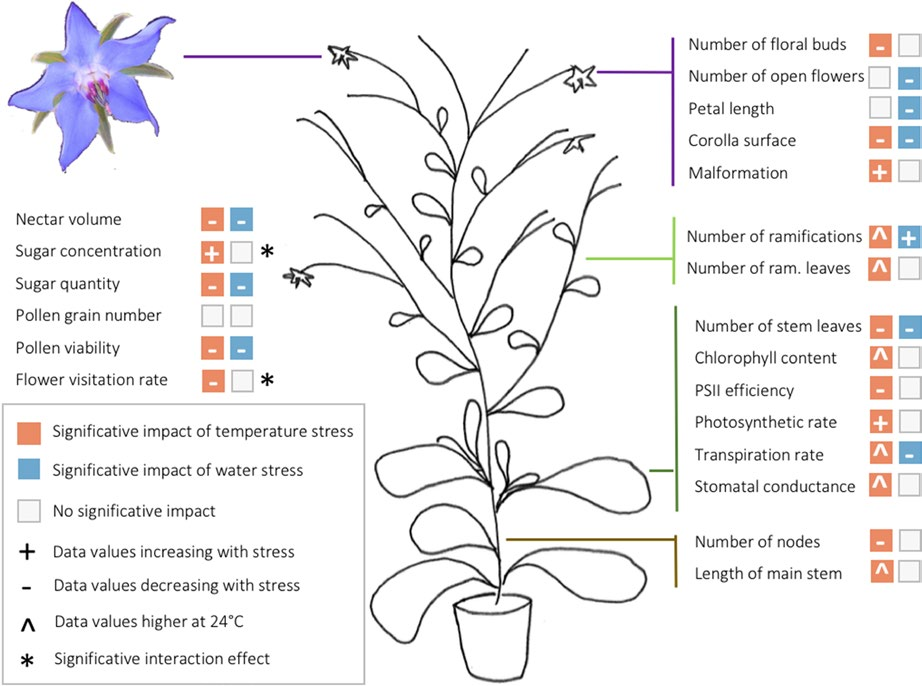
Impact of temperature and water stress on *Borago officinalis* plant functions

Our results showed that both temperature rise and water stress affected *B. officinalis* growth, development, and physiology (Figure 5). Vegetative traits and plant physiology were more affected by temperature, while reproductive growth and flower rewards were affected by both temperature rise and water stress. In *B. officinalis*, the effects of temperature rise and water stress were generally additive as observed for the number of leaves on the main stem, the corolla surface, the nectar production, and the pollen viability. Specific interactions were only observed for the sugar concentration in nectar and the flower visitation rate. The interactions between temperature and water stress were reported to be additive in several plant species, and the combination of both stresses could have a stronger effect on plants than each stress alone depending on the observed parameter (Barnabás et al., 2008; Pandey et al., 2015). However, the combination of temperature and water stresses can also alter plant functions in different ways compared with single stress and have specific effect as observed by Rizhsky et al. (2002) in tobacco.

According to Pandey et al. (2015), both stresses lead to a reduction in photosynthesis. Photosynthesis‐related parameters were mainly affected by temperature during our experiment. Increased temperature influenced light‐dependent photosynthetic reactions more than light‐independent reactions. In our experiment, chlorophyll content and ΦPSII decreased with temperature. Temperature may impair photosynthetic electron transport rate or PSII integrity (Prasad et al., 2008). Temperature rise has been previously shown to negatively affect the light‐dependent phase of photosynthesis (Prasad et al., 2008). However, the light‐independent phase of photosynthesis seemed undisturbed in our experiment; we observed an increase in net photosynthesis with temperature. Stomata remained open under high temperature, allowing gas exchange. E_i_, as well as g_s_, was even higher at 24°C compared to the other temperatures, suggesting that the plant was not stressed at 24°C. WUE increased with temperature, showing that *B. officinalis* deployed mechanisms to cope with high temperatures and limited water loss without reducing photosynthesis. A similar increase in WUE was observed in plants adapted to high temperatures (Tambussi, Bort, & Araus, 2007).

Water stress influenced photosynthesis through plant water status. We observed that *B. officinalis* decreased E_i_ and g_s_ under water stress. A decrease in E_i_ is a common response to water stress; plants close their stomata to limit water loss (Lambrecht, Morrow, & Hussey, 2017; Qaderi et al., 2012). Decreasing g_s_ usually reduces plant photosynthesis (Adejare & Umebese, 2007; Khan et al., 2010). Nevertheless, we observed that the net photosynthetic rate was only slightly affected by water stress in our experiment due to an increase in intrinsic WUE. High WUE has been reported as a strategy to increase resource use efficiency in several species (Gomes et al., 2009; Lambrecht et al., 2017; Quinet, Descamps, Coster, Lutts, & Jacquemart, 2015).

Regarding vegetative development, plant growth was stimulated at 24°C compared to 21°C in *B. officinalis*. Indeed, the length of main stem, the total leaf number, and the ramification length were higher at 24°C compared to the other temperatures. These results suggest that optimum growth temperature was around 24°C for *B. officinalis*. The higher value of several vegetative traits at 24°C could be related to increased gas exchanges and chlorophyll content observed at this temperature. It is well known that photosynthesis activity is correlated with the chlorophyll content (Croft et al., 2017; Fleischer, 1935) and determines the carbon fixation which will be used for vegetative and reproductive growth (Poorter, Anten, & Marcelis, 2013). However, plant growth was impaired at 27°C, with smaller plants and less nodes and leaves on the main stem. Temperature above optimum decreased plant growth in *B. officinalis*, as observed by Qaderi et al. (2012) for *Brassica napus*. The light phase of photosynthesis was affected at 27°C, which could result in a decrease in photosynthesis and sugar production and ultimately in a growth reduction. The CO_2_ import was not affected, but we did not investigate the Rubisco activity neither the sugar production in our study. Salvucci and Crafts‐Brandner (2004) reported that heat stress can inactivate Rubisco in heat‐sensitive plant species, and the carboxylation phase has indeed been reported to be particularly temperature‐sensitive in several plants (Ashraf & Harris, 2013; Salvucci & Crafts‐Brandner, 2004).

Both temperature rise and water stress modified plant architecture, increasing ramification development in *B. officinalis*. Such axillary development was previously reported in response to abiotic constraints (Boutraa & Sanders, 2001; Mathieu et al., 2014) and could be explained by a release of apical dominance. Release of apical dominance is associated with floral transition (Bernier, Kinet, & Sachs, 1981), and our experiment started just at this growth stage. Leaves on the main stem were almost fully developed before the start of the experiment, while leaves on ramifications were initiated during the experiment. During ramification, new leaves were initiated and, simultaneously, leaf senescence was observed on the main stem, primarily under water stress. Leaf senescence is a common response to water stress (Connor & Jones, 1985; Prasad et al., 2008), while temperature can stimulate leaf production up to a species‐specific optimum temperature (Gray & Brady, 2016). We observed that leaf development on ramifications was higher at 24°C than at 21°C or 27°C. The combination of temperature rise and water stress was detrimental to plants above the optimum temperature, in our case, 27°C. Vile et al. (2012) and Prasad et al. (2008) also reported that a combination of high temperature and water stress reduced plant growth and increased leaf senescence, as compared to a single stress.

Regarding reproductive development, high temperatures decreased the number of flower buds, while water stress increased flower abortion, resulting in a low number of open flowers. High temperatures (above 27°C) have been previously shown to cause a reduction in flower number for three *Brassica* species (Morrison & Stewart, 2002) and water stress increased flower abortion in several species (Guo, Chen, Nelson, Cowling, & Turner, 2013; Smith & Zhao, 2016). Kay and Picklum (2013) showed also a lower flower production in drought conditions compared to well‐watered conditions for two *Clarkia* species. Both stresses also affected the flower size, pollen viability, and nectar production in *B. officinalis*. Flower size reduction was previously reported in response to drought. Carroll et al. (2001) observed a reduction of 33% in flower size after 12 days of drought for *Epilobium angustifolium*, and Lambrecht et al. (2017) reported a decrease in flower size of *Leptosiphon androsaceus* in response to dry years. The high water costs of flowering influence flower morphology (De la Barrera & Nobel, 2004). Low pollen viability and male sterility are also common defects in response to both temperature rise and water stress (Erickson & Markhart, 2002; Mesihovic, Iannacone, Firon, & Fragkostefanakis, 2016; Prasad et al., 2008; Smith & Zhao, 2016; Su et al., 2013). Temperature rise and water stress applied during flower morphogenesis particularly affect pollen development, causing abnormalities (Barnabás et al., 2008; Hedhly, 2011; Snider & Oosterhuis, 2011; Zinn et al., 2010). The consequence of temperature rise and water stress on pollen quality will depend on stress intensity and plant species. Sato et al. (2006) observed that under moderate temperature rise (+4°C), pollen production of *Lycopersicon esculentum* was not affected even if pollen viability was reduced (20% compared to 85% for control plants). Under water stress, Su et al. (2013) observed also abnormal anther development and low pollen viability. We observed a reduction in nectar production in response to both stresses in *B. officinalis*. Several studies showed that flower rewards are affected by temperature and water availability. In *Epilobium angustifolium*, Carroll et al. (2001) observed a 60% reduction in nectar volume after 12 days of water stress. Mu et al. (2015) found a 60% reduction in nectar volume for *Saussurea nigrescens* after experimental warming (maximum + 3°C compared to annual mean temperature). A high night temperature can cause a nectar volume decline by increasing the nocturnal plant respiration and the carbohydrate depleting, which would be otherwise allocated to nectar production (Mu et al., 2015). However, in both cases, the sugar concentration was not altered (Carroll et al., 2001; Mu et al., 2015). On the contrary, in our study, the combination of both stresses had a specific effect on sugar concentration as water stress tended to increase the sugar concentration at low temperature and to decrease it at high temperature. In any event, both stresses had additive effects on the total sugar content in nectar per flower, so that the amount of sugars available per flower decreased with the stress intensity.

### 4.2 Do temperature and water stress influence floral traits and flower rewards directly or indirectly?

Floral parameters and flower rewards were affected by both stresses in our experiment. Even if plants maintained growth and physiological status, abiotic stresses had consequences for flower production and development. Reproductive stage is particularly sensitive to abiotic constraints in several species (Korres et al., 2016; Prasad, Djanaguiraman, Perumal, & Ciampitti, 2015; Sage et al., 2015). The effects of abiotic stresses on plant reproduction could be mediated by modifications of plant physiology and vegetative traits but also due to direct effects on flowering and flower development processes.

Our results showed that the number of flower buds and the nectar production were positively correlated with the vegetative development of the main stem and some photosynthesis parameters. Part of the carbohydrates produced by source leaves through photosynthesis could be available for export via the phloem to sink organs such as flowers (Lemoine et al., 2013). According to many studies, up to 80% of photosynthetic fixed carbon can be exported by mature leaves (Lemoine et al., 2013). Restriction in resources could thus lead to a decrease in flower production and to flower abortion. Abortion of floral buds is common when abiotic stresses are applied just before or during anthesis (Prasad et al., 2008; Vile et al., 2012). Regarding nectar production, floral nectaries store large quantity of starch before anthesis which is rapidly converted into sucrose at anthesis for nectar secretion (Roy, Schmitt, Thomas, & Carter, 2017). Thus, a decrease in carbohydrate production or starch transport will limit nectar content. It was reported that drought can induce phloem transport failure affecting access to carbohydrate reserve (Sevanto, 2014). Moreover, Lemoine et al. (2013) reported that the balance between source and sink organs is particularly sensitive to abiotic stresses during reproductive development, so that carbohydrates could be reallocated between source and sink organs. The plant may indeed be regarded as a series of sources and sinks with an overall carbon fixation capacity and several sinks “competing” for the available assimilates (Lemoine et al., 2013). This creates a priority system among sinks, and ramification development could be a major sink as compared with flower development in our experiment. The petal length and the nectar sugar content were indeed negatively correlated with the development of ramifications, confirming the competition between the vegetative and reproductive development in the context of abiotic stresses. The reduction in the reproductive development we observed could thus be partly viewed as an indirect effect of abiotic stresses through modifications of photosynthesis, production of assimilates, and assimilate partitioning between sink organs.

However, reproductive stage is often reported as more sensitive than vegetative traits to abiotic constraints (Hedhly, 2011; Snider & Oosterhuis, 2011; Zinn et al., 2010). Temperature rise and water stress could have more specific effects during this developmental stage. We observed that plant growth was stimulated at 24°C, while reproductive development was already impaired at this temperature. The optimum range of temperature for reproduction is lower than for vegetative growth in several species (Korres et al., 2016; Prasad et al., 2015). Flower morphogenesis was also affected as temperature rise induced floral organ malformations in our experiment. The number of floral organs was modified; for example, flowers developed with only three or four petals. It has been reported that abiotic stresses can reduce the number and the size of floral organs and cause flower deformity or sterility due to altered expression of genes involved in flower morphogenesis (Smith & Zhao, 2016; Zinn et al., 2010). Sterility is induced by abiotic stresses mostly in male floral organ development (Barnabás et al., 2008; Hedhly, 2011; Smith & Zhao, 2016; Snider & Oosterhuis, 2011; Zinn et al., 2010). We observed increasing pollen grain abnormalities with temperature rise and water stress, decreasing the number of mature pollen grains. Defects appear particularly during meiosis, tapetum development, anthesis, dehiscence, and fertilization (Smith & Zhao, 2016). These modifications can alter the chemical composition of pollen, a parameter that modulates plant–pollinator interactions (Muth, Francis, & Leonard, 2016) and plant reproductive success (Zinn et al., 2010). The female organs are generally not as susceptible as the male organs to abiotic stresses (Smith & Zhao, 2016), but female gametophyte fertility and seed development could be affected by abiotic stresses (Hedhly, 2011; Su et al., 2013). Heat and drought stresses reduced the sexual organ fertility and the flower‐reward content in our study, but the final impact on reproductive success needs further investigations.

### 4.3 Do these stresses modify flower visitation by insects?

Overall, we observed that stressed flowers were less visited than nonstressed flowers. Insects visited mainly flowers for nectar and pollen collection (Nicolson, 2011), and modifications in flower rewards may explain the observed visitation rates. As previously mentioned, flower rewards were impacted by the temperature and water stresses. The visitation rate was positively correlated with the nectar production in *B. officinalis*. Without stress, flowers produced a high nectar sugar quantity (3.9 mg/flower) and were more visited compared to flowers under temperature rise and water stress, which produced a lower nectar sugar quantity (1.3 mg/flower). Bees can modify their foraging behavior in response to nectar modification, and bees visited more flowers on plants that produced higher nectar quantity than others (Blarer, Keasar, & Shmida, 2002; Cartar, 2004; Chittka, Gumbert, & Kunze, 1997; Dreisig, 2012). However, at 27°C, we observed a higher visitation rate for plants under water stress compared to well‐watered plants. One could hypothesize that the low nectar volume led insects to visit more flowers on the same plant to collect a minimum volume of nectar. The sugar concentration in nectar was about 60% at 27°C, and it is known that bumblebees discriminate between small differences in nectar concentration and prefer sugar concentrations higher than 40% (Cnaani et al., 2006). Even under stress conditions, the nectar production of *B. officinalis* was similar or higher as compared with other attractive bee‐pollinated species (Baude et al., 2016; Somme et al., 2016), suggesting that *B. officinalis* remains an interesting nectar source whatever the environmental conditions.

In addition to nectar, pollen amount and quality also affect pollinator foraging behavior (Cook, Awmack, Murray, & Williams, 2003). Although the amount of pollen per flower was not affected in our study, the pollen viability decreased with the stress intensity. Pollen of low viability has most probably also a lower quality for insects (Muth et al., 2016). The pollen viability may thus also explain the flower visitation in our experiment. Likewise, without stress, flowers produced pollen with high viability compared to stressed flowers, mainly at 27°C, which produced pollen with low viability. The effects of temperature and water stress on chemical composition of pollen need further investigations. Indeed, pollen chemical composition influences bee visitation behavior (Cook et al., 2003; Muth et al., 2016; Somme et al., 2014; Vanderplanck et al., 2014). The relative contribution of nectar and pollen to explain the decrease in visitation rates remains to be studied.

The flower rewards are not the only parameters that drive the flower visitation by insects, and other floral traits are linked to flower attractiveness (Fowler, Rotheray, & Goulson, 2016). Temperature and water stresses affected other parameters such as floral morphology. Firstly, we observed that stressed flowers had a reduced size even though this parameter was not correlated with flower visitation in our experiment. On the contrary, Stanton and Preston (1988) showed that flower size was correlated with pollinator visitation in *Raphanus sativus*. Secondly, we observed flowers with meristic or homeotic modifications of the floral organs. Modifications such as abnormal stamen or nectary development impair flower‐reward accessibility. Modifications of floral morphology could alter pollinator choice, leading to patch abandonment (Chittka, Thomson, & Waser, 1999). The impact of floral morphology on *B. officinalis* pollination will be further investigated.

In conclusion, we observed that although *B. officinalis* growth and development were more affected by temperature rise than water stress, both stresses had mainly additive effects. Despite *B. officinalis* developed physiological mechanisms that limit the negative impact of these abiotic stresses, floral traits and flower rewards were substantially altered by these stresses, compromising pollinator attractiveness and potentially plant reproductive success.

## CONFLICT OF INTEREST

There is no conflict of interest.

## AUTHOR CONTRIBUTION

C. Descamps, M. Quinet, and A.‐L. Jacquemart designed the experiments. C. Descamps, M. Quinet, and A. Baijot performed field and laboratory experiments. M. Quinet and A.‐L. Jacquemart supervised the study. C. Descamps and M. Quinet wrote the manuscript. All authors revised the draft manuscript and read and approved the final manuscript.
